# Alcohol Consumption and the Neoplastic Progression in Barrett's Esophagus: A Systematic Review and Meta-Analysis

**DOI:** 10.1371/journal.pone.0105612

**Published:** 2014-10-09

**Authors:** Zhifeng Lou, Haibo Xing, Da Li

**Affiliations:** 1 The department of stomatology, Sir Run Run Shaw Hospital Affiliated to Zhejiang University School of Medicine, Hangzhou, Zhejiang, P. R. China; 2 The department of intensive care unit, Xiasha Hospital, Hangzhou, Zhejiang, P. R. China; 3 Department of Medical Oncology, Sir Run Run Shaw Hospital Affiliated to Zhejiang University School of Medicine, Hangzhou, Zhejiang, P. R. China; Shiga University of Medical science, Japan

## Abstract

**Purpose:**

In the developed countries, the incidence of esophageal adenocarcinoma (EAC) is increasing over recent decades. The purpose of this meta-analysis was to arrive at quantitative conclusions about the contribution of alcohol intakes and the progression of Barrett's esophagus.

**Methods:**

A comprehensive, systematic bibliographic search of medical literature published up to Oct 2013 was conducted to identify relevant studies. A meta-analysis was conducted for alcohol consumption on the Barrett's esophagus progression.

**Results:**

A total of 882 cases in 6,867 individuals from 14 observational studies were indemnified in this meta-analysis. The result of this current meta-analysis, including 10 case-control and 4 cohort studies, indicated that alcohol consumption was not associated with the neoplastic progression in Barrett's esophagus (RR, 1.17; 95% CI, 0.93–1.48). When stratified by the study designs, no significant association was detected in either high vs low group or ever vs never group.

**Conclusions:**

Alcohol drinking is not associated with risk of neoplastic progression in Barrett's esophagus. Further well designed studies are needed in this area.

## Introduction

The incidence rate of Barrett's esophagus in the patients with gastroesophageal reflux disease (GERD) was 10% to 15% [1]. Endoscopic surveillance has been recommended for patients with Barrett's esophagus because of its association with esophageal adenocarcinoma (EAC). In the developed countries, the incidence of EAC increased over recent decades [2], [3]. Barrett's esophagus, which is a condition of the distal esophagus in which the normal stratified squamous epithelium is replaced by specialised intestinal metaplasia, is a recognized precursor of EAC. The metaplasia in Barrett's esophagus patients accumulates genetic alterations and can progress through dysplasia to EAC.

Factors that may be associated with an increased risk of Barrett's esophagus or EAC were examined, including: age, gender, ethnicity, smoking, alcohol use, Barrett segment length, hiatal hernia presence and size, *Helicobacter pylori* status, presence and duration of GERD symptoms, and proton pump inhibitor (PPI) use and duration. However, the risk factors of the neoplastic progression in the Barrett's esophagus remained unclear. Alcohol, as a risk factor of several kinds of cancers [4], was reported to be associated with the incidence of both Barrett's esophagus and EAC. However, the effect of alcohol intake on the neoplastic progression in Barrett's esophagus was still unclear.

Meta-analysis is a useful statistical tool to pool the relevant studies together and gain a more powerful conclusion. The meta-analysis was also used in the search for potential causes of EAC. For instance, based on a combination of 4 cohorts and 31 case-control studies, Salehi M et al found that low levels of red and processed meat consumption and higher levels of fish intake might reduce esophageal cancer risk [5]. Although early studies made several attempts to summarize the epidemiologic evidence on alcohol drinking and the progression of Barrett's esophagus, to our knowledge, no meta-analyses have ever been conducted in a standardized manner. To quantitatively assess the potential relationship, we conducted a comprehensive meta-analysis of case-control and cohort studies conducted between 1993 and 2013.

## Methods

### Search Strategy and Inclusion Criteria

As a meta-analysis of observational studies, this current meta-analysis was conducted according to the PRISMA guidelines and MOOSE guidelines [6],[7]. A systematic search of Pubmed and Embase databases was conducted for relevant literature published up to Oct. 2013 with the key words “esophag*”, “adenocarcinoma”, “carcinoma”, “cancer” in combine with “Barrett's esophagus” and “alcohol”, “drink” or “wine” or “beer”. No language or any other restrictions were set in the search strategy. In addition, we also manually searched the reference lists to detect additional eligible studies. When different articles from the same dataset were obtained, only the most recent study with available data was included in the meta-analysis. The contact with the corresponding author of certain article was conducted when more data was required.

Studies satisfying the following criteria were included in the observational meta-analysis: (1) the association between alcohol consumption and risk of Barrett's esophagus progression; (2) a cohort or case-control study design was obtained; (3) the value of relative risk (RR), odds ratio (OR) with 95% confidence intervals (CI) or enough data to calculate them were reported.

### Data Extraction and Assessment of Study Quality

Two reviewers (ZL and HX) independently extracted the data and any discrepancy was checked again and resolved through discussion. The following data was extracted from each article: name of the first author, publishing year, study design, study site, age and gender of participants, type and amount of cases, adjustments of the confounding factors, and the OR/RR value with 95% CI.

Quality assessment that was performed by two reviewers back to back and any disagreement was discussed with the third reviewer. Considering the observational study design of the included studies, the Newcastle-Ottawa Scale (NOS) was obtained to assess the methodological quality of the included studies [8]. It assessed the selection, comparability and exposure of a case-control study, while the selection, comparability and outcome of a cohort study. A maximum of 9 stars was scored for a study and the study with over 6 stars would be regarded as relative high quality.

### Statistical Methods for the Meta-analysis

Homogeneity of RR across studies was assessed by using the Cochrane Q statistic and I^2^ statistic. When *P* for the heterogeneity <0.1 and *I^2^*>50%, the interstudy heterogeneity would be considered statistically significant. Both OR and RR were reported in the included studies and the OR was obtained to approximate RR in this meta-analysis. When both crude and adjusted OR/RR values were offered in the article, only the adjusted ones were adopted for the meta-analysis. A random-effects model was obtained to estimate the pooled effects, thus it would provide a more conservative conclusion. The effects of alcohol drinking on the incidence of Barrett's esophagus progression were measured with the OR with 95% CI. The sensitivity analyses were conducted to detect the robustness of the conclusions by two independent methods. Firstly, we conducted a sensitivity analysis to investigate the influence of a single study on the overall risk estimate by omitting one study in each turn. Secondly, we excluded the studies with a relative lower methodological quality and assess the effect of alcohol consumption and risk of progression of Barrett's esophagus.

We constructed a funnel plot with logRR and their SEs of logRR by visual inspection to assess the potential publication bias. Besides, potential publication bias was also assessed by both Begg's rank correlation test [9] and Egger linear regression test [10] at the p<0.10 level of significance. All analysis was performed using STATA version 12.0 statistical software (Stata Corp LP, College Station). A p value <0.05 was considered as statistically significant.

## Results

### Identification and Selection of Studies

**Figure 1:**
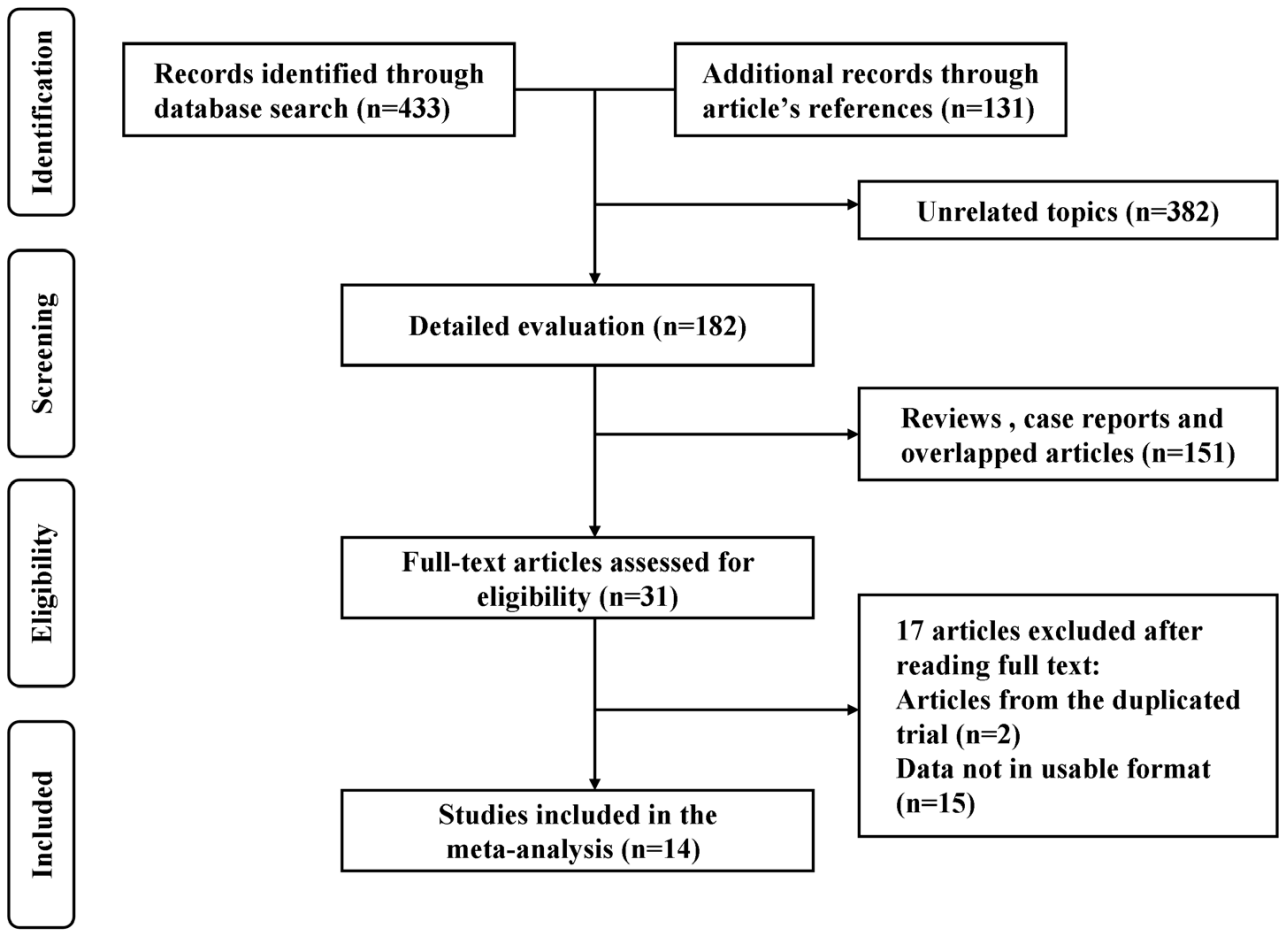
Flow chart of the literature search. A totaol of 14 observational studies (10 case-control and 4 cohort studies) were indemnified in this meta-analysis.

A total of 433 articles (198 from Pubmed and 235 from EMBASE) were identified from the electronic database searching. Besides, 131 more records were identified through consulting the reference lists of the relevant reviews and articles. After excluding 382 articles with unrelated topics, a total of 182 records were detailed evaluated. Among the 182 articles, 31 full-texts were assessed for eligibility after removing 151 articles (reviews, case reports and overlapped articles). Subsequently 2 articles were duplicated reports from previous data and 15 ones in which the data not in usable format were excluding from the inclusion and in final, a total of 14 studies were included for the quantitative synthesis [11]–[24]. Figure 1 demonstrated the selection of studies.

### Study Characteristics and Quality

A total of 882 cases and 6,867 individuals in 14 observational studies were indemnified in this current meta-analysis. All the detailed characteristics of each included study were presented in Table 1. The included studies were published between 1993 and 2013. A total of 4 cohort studies and 11 case-control studies were identified in this current meta-analysis. Among all the included studies, 8 studies were in European, 5 in Americas and 1 in Asia. The age, gender distribution, categories of alcohol consumption, and adjustments of confounding factors were demonstrated in Table 1.

**Table 1:** Characteristics of eligible studies.

Study; Year	Study design	Site	Age (Year)	Gender (Percent)	Type and No. of cancer	No. of control/non-cancer	Adjustment or matched	Exposure Definition	Quality assessment criteria 1			
									Selection	Selection	Selection	Selection
Lukić M, et al 2010 [11]	Retrospective Hospital CC	Croatia	17–83	NA	EAC 20	20	Age, gender, somking	4, No, 0.3dL/W,0.5dL/d, >1dL/d	**	*	***	6
Anandasabapathy S, et al 2007 [12]	Retrospective Hospital CC	USA	25–85	M 76.15	HGD/EAC 35	74	Sex, men, hiatal hernia size, Barrett length, GERD symptom, GERD duration, H. pylori absent, race, smoking	2, 1–6 drinks/wk, 7 drinks/wk	**	**	***	7
Bani-Hani KE, et al 2005 [13]	Retrospective Hospital CC	China	2–94	M 56	EAC 44	553	Age ≥60 yr, male sex, NSAIDs, EAC, stricture	2, Non-alcohol use, regular alcohol use	***	**	***	8
Coleman HG, et al 2012 [14]	Retrospective Population Cohort	UK	62	M 62.80	HGD/EAC 117	3050	Age groups, sex, presence of low-grade dysplasia, income deprivation quintile, Barrett segment length and presence of reflux symptoms.	3, None, 10 units/week, 10 units/week	***	**	***	8
Anderson LA, et al 2007 [15]	Retrospective PopulationCC	UK	63.4	M 82.86	EAC 227	224	Age, gender	2, Never, ever	**	**	**	6
de Jonge PJ, et al 2006 [16]	Prospective Hospital CC	Netherlands	62.5	M 76	EAC 91	244	Age, gender, educational level, smoking, and reflux symptoms	3, Never, former, current	***	**	***	8
Gatenby PA, et al 2008 [17]	Retrospective Hospital Cohort	UK	63.4	M 62.5	HGD/AC 63	1002	Age, gender	2, High, low	***	*	**	6
Achkar JP, et al 1995 [18]	Retrospective Hospital CC	USA	63.5	M 96.47	EAC 85	85	Age, sex, tobacco smoking	2, Alcohol abuse, non alcohol abuse	**	**	**	6
Sikkema M, et al 2011 [19]	Prospective Population CC	Netherlands	20–86	M 74	HGD/EAC 26	687	Age and gender	3, Never,former,current	***	**	***	8
Jung KW, et al 2011 [10]	Retrospective Population Cohort	USA	61.94	M 69.61	HGD/EAC 26	329	Age	3, None, current, past	**	*	**	5
Casson AG, et al 2005 [21]	Prospective Hospital CC	Canada	NA	M 77.35	EAC 56	125	Age, gender, smoking	2, Ever, never	*	*	**	4
Gray MR, et al 1993 [22]	Retrospective Hospital CC	UK	31–83	M 83.95	EA 23	58	Age, sex	2, Ever, never	**	**	**	6
Olliver JR, et al 2005 [23]	Prospective Hospital CC	UK	30–86	M 72.97	EA 24	50	Age, sex	2, Ever, never	**	*	**	5
Hardikar S, et al 2013 [24]	Prospective Population Cohort	USA	61.2	M 81.3	EA 45	366	Age, gender, WHR, NSAID use and cigarette smoking	4,0, 0–1, 1–3, >3 drinks/day	***	**	**	7

NA: not available; CC: case-control study; LGD: low-grade dysplasia; EAC: esophageal adenocarcinoma; NASID: non-steroids antiinflammatory drugs; GERD: gastroesophageal reflux disease; WHR: waist-to-hip ratio; BMI: bady mass index; BE: Barrett esophagus; M: male.

1 The study quality was assessed by Newcastle-Ottawa Scale.

The NOS were obtained to assess the selection, comparability and exposure of the case-control study, while the selection, comparability and outcome of the cohort study. All the scores of each part in the evaluation of all the studies were displayed in Table 1. Eleven in nine studies were in relative high quality (over 6 stars) and the mean NOS score was 6.43 stars (standard deviation: 1.28).

### Alcohol Consumption and Neoplastic Progression in Barrett's Esophagus

**Figure 2:**
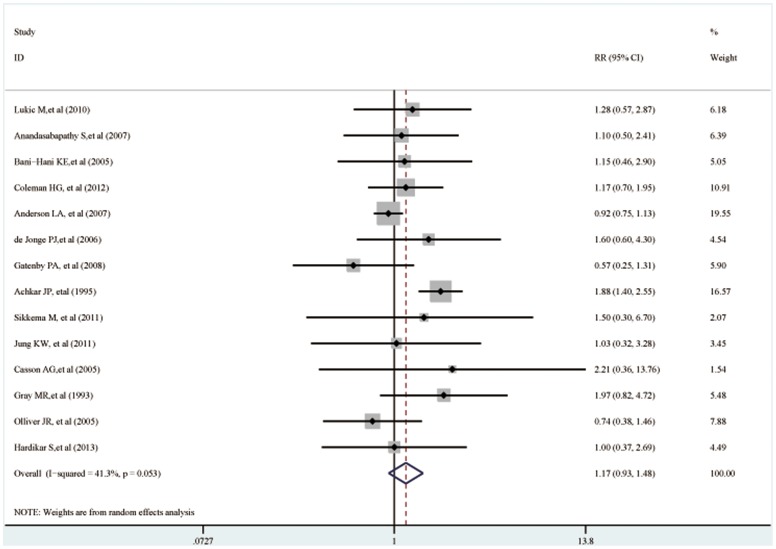
Forest plot of the association between alcohol drinking and risk of Barrett's esophagus progressiom.

Figure 2 showed the pooled estimation of the alcohol consumption and neoplastic progression of Barrett's esophagus in a random-effects model. In this meta-analysis, alcohol intake was not associated the incidence of HGD or EAC (RR, 1.17; 95% CI, 0.93–1.48). Besides, no significant association was detected in neither cohort studies (n = 4; RR, 0.97; 95% CI, 0.67–1.42) nor case-control studies (n = 10; RR, 1.20; 95% CI, 0.96–1.50). In the two data source subgroups, no significant association was in neither population-based group (n = 6; OR, 0.99; 95% CI, 0.87–1.13) nor hospital-based group (n = 9, OR, 1.27; 95% CI, 0.92–1.75). When stratified by the neoplastic progressions, no significant association between alcohol consumption and HGD/EAC (n = 5; RR, 1.02; 95% CI, 0.72–1.45) nor EAC (n = 11; RR, 1.13; 95% CI, 0.72–1.45) was detected. When the geographicical distributions of the included studies were considered, the studies that conducted in the Europe (n = 8; RR, 1.00; 95% CI, 0.88–1.13), and the Asia (n = 1; RR, 1.14; 95% CI, 0.95–1.38) showed no statistically significant results. However, alcohol intake was associated with increased risk of HGD or EAC in the Americas (n = 5; RR, 1.66; 95% CI, 1.28–2.15). No significant association was detected in neither high vs low group (n = 6, RR, 1.00; 95% CI, 0.85–1.18) nor ever vs never group. All the results of the subgroup analysis were presented in Table 2.

**Table 2:** Subgroup analysis of alcohol consumption and BE progression with combined RR.

	Subgroups	No. of studies	Summary Effect			Study Heterogeneity	
			RR	95% CI	P Value	I^2^, %	P Value
Study design	Cohort	4	0.97	0.67 to 1.42	0.887	0.00%	0.554
	Case-control	10	1.2	0.96 to 1.51	0.115	50.80%	0.026
	Population-based	6	0.99	0.87 to 1.13	0.932	38.80%	0.0862
	Hospital-based	9	1.267	0.92 to 1.75	0.110	0.00%	0.932
	Prospective	6	0.980	0.86 to 1.12	0.767	0	0.776
	Retrospective	8	1.31	0.98 to 1.75	0.064	30.30%	0.186
Design	High vs low	6	1.002	0.85 to 1.18	0.978	0.00%	0.794
	Ever vs never	9	1.252	0.91 to 1.73	0.175	67.30%	0.001
End	HGD and EAC	5	1.017	0.72 to 1.45	0.926	0.00%	0.658
	EAC	11	1.129	0.91 to 1.40	0.272	49.90%	0.025
Site	Europe	8	1.00	0.88 to 1.13	0.938	0	0.494
	Americas	5	1.656	1.27 to 2.15	<0.001	0	0.484
	Asia	1	1.142	0.95 to 1.38	0.766	-	-

HGD: high-grade dysplasia; EAC: esophageal adenocarcinoma; RR, relative risk; CI, confidence interval.

### Heterogeneity, Sensitivity analysis and Publication bias

The heterogeneity was significant when all the 14 studies were pooled in the meta-analysis (*I^2^*, 41.3%; *P* = 0.063). We tried to explore the source by excluding the included studies one by one and re-count the heterogeneity. When one study [18] was removed from the meta-analysis and the heterogeneity became non-significant (*I^2^*, 9.2%; *P* = 0.769). The result of this current meta-analysis didn't change when that study was excluded (RR, 0.99; 95% CI, 0.85–1.16).

A one-way sensitivity analysis was conducted and there was little change in the quantitative summary measure of RR or 95% CI. Although one studies [15] seemed to slightly influence the results, there was no change to the direction of effect, when anyone study was excluded. Besides, we just included the articles with a relative high quality (over 6 stars NOS score in the meta-analysis); however, no significant association was detected neither (n = 11; RR, 1.21; 95% CI, 0.93–1.58). A significant heterogeneity should be noted as well (*I^2^* = 50.1%, *P* = 0.029).

Evidence of publication bias for studies in this current meta-analysis wasn't noted in symmetrical funnel plot on visual inspection (Figure S1). Both Begg's graph and Egger's test were obtained to detect the potential publishing bias. No significant publication bias was found in this current meta-analysis (Begg's test, *P* = 0.381; Egger's test, *P* = 0.645).

## Discussion

A total of 882 EAC cases in 6,867 individuals from 14 observational studies were identified in this meta-analysis. The result of this meta-analysis, including 10 case-control and 4 cohort studies, indicated that alcohol consumption was not associated with the neoplastic progression in Barrett's esophagus. Meanwhile, this result was demonstrated in the most subgroup analyses by study design, study sites, end points. However, the studies conducted in the Americas showed that alcohol drinking increased the neoplastic risk. When stratified by the study designs, no significant association was detected in either high vs low group or ever vs never group. The heterogeneity was not significant when all the 14 studies were pooled in the meta-analysis. Publication bias was not detected in the meta-analysis. The results of the sensitivity analysis suggest that the conclusions of this study were quite robust.

EAC is now a more and more serious problem in the entire world [25]. It affects the life quality of the patients with EAC. While risk factors for the development of EAC in the general population have been well investigated, it is largely unclear which kinds of patients with Barrett's esophagus have an increased risk for malignant progression. It is important to detect the harmful or protective factors for the EAC in the Barrett's esophagus patient and it might help in the primary prevention. The realization of the relationship between the modifiable epidemiological factors and neoplastic progression in Barrett's esophagus would provide a more effective strategy for the cancer prevention in the future. Alcohol consumption, which was related with the incidence of both the Barrett's esophagus and EAC, was considered to play a role in the progression from Barrett's esophagus to the EAC. However, inconsistent evidence exists regarding the effect of alcohol consumption on the neoplastic progression in Barrett's esophagus.

In this meta-analysis, we found that alcohol consumption is not associated with progression of Barrett's esophagus. This result supports the conclusions of several previous studies. In 2003–2004, a prospective, multicenter cohort study including 713 patients with Barrett's esophagus was conducted. After 4 years of follow-up, duration of Barrett's esophagus of ≥10 years, length of Barrett's esophagus, esophagitis, and LGD were significant predictors of progression to HGD or EAC; however, alcohol intake was not associated with the progression of Barrett's esophagus (RR, 1.5; 95% CI, 0.3–6.7). In a hospital-based case-control study in which 91 cases with EAC and 244 controls with histologically confirmed Barrett's esophagus (>2 cm) with no dysplasia or low-grade dysplasia were included, current alcohol use was not associated the incidence of EAC (RR, 1.6; 95% CI, 0.6–4.3) [16]. The data from patients with Barrett's esophagus identified from the population-based Northern Ireland Barrett's esophagus register, diagnosed between 1993 and 2005 with specialized intestinal metaplasia (n = 167) was analyzed and the result showed alcohol intake was not associated with increased risk of HGD or EAC after adjusting for several confounding factors (RR, 0.82; 95% CI, 0.41–1.62). When the subgroup analyses stratified by the study designs was conducted, the associations between alcohol consumption and Barrett's esophagus progression wasn't detected in neither case-control nor cohort studies. As we know, the cohort study design could avoid the potential recall bias and would provide more credible conclusions. A consistent result is obtained in both the case-control and cohort studies and it suggests that the result of this meta-analysis is quite credible.

However, the studies conducted in the Americas showed that alcohol drinking was a risk factor of the progression of Barrett's esophagus. The geographical differences, the diet diversity and ethnic and genetic disparity are the possible reasons. Alcohol consumption, which was classified as beer, liquor and wine intake, might demonstrate different effect in the development of Barrett's esophagus. The difference of the drinking habits in each region might cause the results. Besides, the relatively small number of studies included in the subgroup analyses (5 in the Americas and 1 in the Africa) might lead to the instability of the conclusions. When stratified by the end points, no significant association was detected in either HGD or EAC or just EAC. It suggests that alcohol might be not associated in any stage of the Barrett's esophagus progression.

To our best knowledge, this is the first meta-analysis involving the relationship between alcohol consumption and risk of development of Barrett's esophagus. There are some strengths of this work. In the literature search, a large number of subjects were evaluated for the detection of the effect of the neoplastic risk in Barrett's esophagus associated with alcohol drinking. Besides, through two independent methods, the publication bias wasn't significant. The results of the sensitivity analysis suggest that the conclusions of this study were quite robust. These above results demonstrated that the conclusions of this meta-analysis were quite persuasive.

Despite these advantages mentioned above, some limitations of the current meta-analysis should be acknowledged. First, the definitions of the case groups were not uniformly defined. Both the patients without higher alcohol intake or ever alcohol intake were obtained as the controls in the included studies. This would produce potential misclassification bias. While the subgroup analyses showed that no different results were detected and thus this might produce no serious problem. Secondly, even the categories of alcohol consumption are reported in some studies; however, the data were varied and broad. Thus, we were unable to determine the dose-response association between alcohol consumption and progression of Barrett's esophagus. Third, it has been argued that because meta-analyses of observational studies may produce very precise, but spurious, results, a statistical combination of these data should not be the prominent component. However, considering that as an etiology exploring nature of this study, the pooled results of the observational studies would also provide certain improvement of the knowledge of the neoplastic development. The end, a combination of prognostic factors has been suggested to be required to define subgroups of patients at an increased risk of progression. However, the data of the included studies reported no sufficient data for an advanced research, which demonstrated the potential breakthrough. The forth, the understanding of the Barrett's esophagus has developed in the recent years. The old conception says that although several different kinds of columnar epithelium can be found in the lower esophagus, it is only specialized intestinal metaplasia (SIM), distinguished by the presence of goblet cells, which increased the cancer risk. However, nowadays *recent studies* revealed that cardiac-type mucosa, columnar lined esophagus without goblet cells, also have the posibility of cancer development. With the development of understanding of Barrett's esophagus, the older papers might provide results based on inaccurate definitions and thus the bias could not be ignored. Advanced well-designed studies based on both endoscopic results and histocytology are wanted in the future.

In conclusion, on the basis of epidemiologic evidence, we found that alcohol consumption was not a neoplastic risk in Barrett's esophagus. To get a more definitive conclusion, further pooled analyses with more complete raw data or prospective cohort studies with larger sample size, well controlled confounding factors and longer duration of follow-up are needed in this area.

## Supporting Information

Figure S1 **The funnel plot of all the included studies.** Evidence of publication bias for studies in this current meta-analysis wasn't noted in symmetrical funnel plot on visual inspection.
(TIF)

Checklist S1 **PRISMA checklist.**
(DOC)
