# Supplementary figures and images for: Alcohol Consumption and the Neoplastic Progression in Barrett's Esophagus: A Systematic Review and Meta-Analysis
Source: PLoS One. 2014 Oct 9;9(10):e105612. doi: 10.1371/journal.pone.0105612 (PMC4191954; doi:10.1371/journal.pone.0105612)

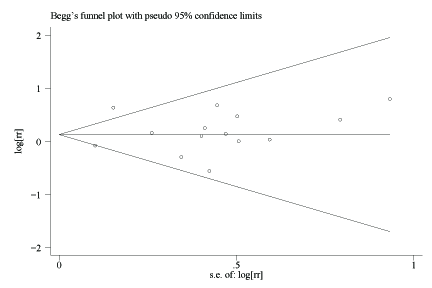

Supplement: Figure S1 — The funnel plot of all the included studies. Evidence of publication bias for studies in this current meta-analysis wasn't noted in symmetrical funnel plot on visual inspection. (TIF) [file pone.0105612.s001.tif]
